# Human Urinary Kallidinogenase Reduces Lipopolysaccharide-Induced Neuroinflammation and Oxidative Stress in BV-2 Cells

**DOI:** 10.1155/2019/6393150

**Published:** 2019-07-24

**Authors:** Zhongyan Zhao, Zhiyu Xu, Tao Liu, Shixiong Huang, Huai Huang, Xiaoyun Huang

**Affiliations:** ^1^Department of Neurology, Hainan General Hospital, Haikou 570311, China; ^2^Department of Critical Care Medicine, Hainan General Hospital, Haikou 570311, China; ^3^Neurorehabilitation Dept. 2, Guangzhou General Hospital of Guangzhou Military Command of PLA, Guangzhou 510120, China; ^4^Department of Neurology, The Affiliated Houjie Hospital, Guangdong Medical University, Dongguan 523945, China

## Abstract

Migraine is one of the most common neurological disorders which poses significant socioeconomic burden worldwide. Neuroinflammation and oxidative stress both play important roles in the pathogenesis of migraine. Human urinary kallidinogenase (UK) is a tissue kallikrein derived from human urine. Increasing evidence suggests that UK may protect against ischemic stroke, but UK's treatment potential against migraine remains to be explored. Immortal BV-2 murine microglial cells were treated with UK (125 nM, 250 nM, and 500 nM) and then given lipopolysaccharides (LPS, 1000 ng/mL). Cell viability of BV-2 cells was tested by the CCK-8 assay. Expressions of tumor necrosis factor-*α* (TNF*α*), prostaglandin E2 (PGE2), interleukin-6 (IL-6), and interleukin-1*β* (IL-1*β*) were examined with the ELISA method and western blot. Intracellular reactive oxygen species (ROS) and malondialdehyde (MDA) were measured to determine oxidative stress. Our results showed that LPS administration increased the levels of proinflammatory cytokines (TNF*α*, PGE2, IL-6, and IL-1*β*) and oxidative stress (ROS and MDA) when compared with the control group and decreased significantly upon introduction with UK. Taken together, UK treatment reduced LPS-induced neuroinflammation and oxidative stress in a dose-dependent manner, which might be a potential treatment of migraine.

## 1. Introduction

Migraine is one of the most common neurologic disorders and is a major cause of disability worldwide [1]. It is a kind of unilateral pulsating headache with clinical symptoms of nausea, vomiting, phonophobia, and photophobia [2]. Antiepileptic drugs (AEDs), beta-blockers, and tricyclic anti-depressants are commonly used agents in the preventive treatment of migraine [3]. Since migraine poses significant socioeconomic burden, looking for new therapies has become an urgent international health priority [4].

The hypothesis that migraine is a neurovascular disorder and the headache is caused by dilation of cerebral and meningeal arteries has been well established [5]. However, the exact mechanism of migraine is still not well understood. Many research studies have proved that neuroinflammation and oxidative stress played important roles in the pathogenesis of this disease [6, 7].

The kallikrein-kinin system (KKS) consists of kinins, kallikreins, kininogens, and kinin receptors. Kinin plays its role by binding to the receptor, resulting in neuroprotective effect [8]. However, kinin could not be used as a drug because of its short half-life [9]. In contrast, kallikrein has much more amount in plasma. Accumulated studies have reported the function of tissue kallikrein on antiapoptotic, antioxidant, and antiexcitotoxic properties, suggesting that tissue kallikrein could be an effective therapy for neurological disorders [10]. Human urinary kallidinogenase (UK) is a tissue kallikrein derived from human urine, cleaving kininogen to release bradykinin [11]. UK has been considered to be a positive regulatory substance in the kallikrein-kinin system by increasing kallikrein. Recently, UK has been widely used in China to treat ischemic stroke patients [12]. However, UK's treatment potential in migraine has not yet been evaluated.

In the present study, we evaluated a UK's effects on neuroinflammation and oxidative stress in LPS-stimulated BV-2 cells.

## 2. Materials and Methods

### 2.1. Cell Culture

Immortal BV-2 murine microglial cells were cultured as described [13, 14]. BV-2 cultures were treated with UK (125 nM, 250 nM, 500 nM, 750 nM, and 1000 nM) for 12 h and then with LPS (125 ng/mL, 250 ng/mL, 500 ng/mL, 750 ng/mL, and 1000 ng/mL, LotL2880, O55:B5, Sigma-Aldrich, St. Louis, MO, USA) for another 12 h. BV-2 cells were cultured at 37°C in Dulbecco's modified Eagle's medium (DMEM) with 1% of 100 U/mL of penicillin/streptomycin and 5% fetal bovine serum. All reagents were purchased from Gibco Thermo Fisher Scientific Inc. (MA, USA). UK was purchased from Techpool Bio-Pharma Co. Ltd., Canton, China.

### 2.2. CCK-8 Assay for Cell Viability

The effects of UK and LPS on BV-2 cell viability were detected by the CCK-8 assay [15]. In brief, cells were cultured on a 96-well plate at a density of 1 × 10^4^ per well for 24 h and then administrated with UK (125 nM, 250 nM, 500 nM, 750 nM, and 1000 nM) for 12 h, or with LPS (125 ng/mL, 250 ng/mL, 500 ng/mL, 750 ng/mL, and 1000 ng/mL) treatment for another 12 h. Then, the cells were incubated at 37°C for 2 h and the absorbance values of the samples were measured at 450 nm by a multifunctional microplate reader (SpectraMax M5, Sunnyvale, CA, USA).

### 2.3. Enzyme-Linked Immunosorbent Assay (ELISA)

The cells and the samples were stored at −80°C until analysis. We measured the concentration of tumor necrosis factor-*α* (TNF*α*), prostaglandin E2 (PGE2), interleukin-6 (IL-6), and interleukin-1*β* (IL-1*β*) with the ELISA method, which have been administrated with UK. The assays were performed using commercially available ELISA kits (Thermo Scientific, USA) according to the manufacturer's instructions. The total protein concentration was determined using the BCA Protein Assay kit (Thermo Scientific, USA). The absorbance of the samples was detected with a multifunctional microplate reader (SpectraMax M5, Sunnyvale, CA, USA).

### 2.4. Measurement of Oxidative Stress

Intracellular reactive oxygen species (ROS) was measured using the fluorescent probe 2,7-dichlorofluorescein diacetate (DCFH-DA) [16]. Another indicator of oxidative stress malondialdehyde (MDA) was detected with commercial kits as described previously [17].

### 2.5. Western Blot Analysis

BV-2 cells were washed three times with cold PBS, and the proteins were quantified with the BCA assay. Afterward, the PVDF membranes were incubated with primary antibodies at 4°C overnight and incubated with horseradish peroxidase-conjugated secondary antibodies for 1 h (anti-rabbit/anti-mouse IgG). Primary antibodies used were listed as follows: GAPDH as the loading control (1 : 1000, Cell Signaling Technology), TNF*α* (1 : 1000, Cell Signaling Technology), IL-6 (1 : 4000, Biosource), and IL-1*β* (1 : 2000, Rockland). The densitometric values of the bands were measured using the ImageJ software (National Institutes of Health, USA). The ratio relative to GAPDH for each band was calculated.

### 2.6. Statistical Analysis

SPSS 16.0 for Windows (SPSS Inc., Chicago, IL, USA) was used to carry out the statistical analyses. One-way ANOVA and Student's *t*-test were used for comparisons between groups. The data were expressed as the mean ± SEM, and differences were considered statistically significant at *p* < 0.05.

## 3. Results

### 3.1. Cell Viability

**Figure 1:**
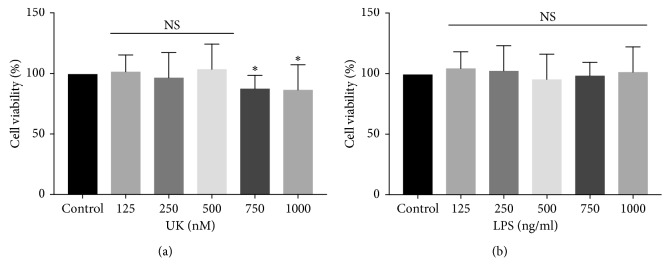
Cell viability of BV-2 microglial cells treated with UK and LPS. Cell viability of BV-2 cells was tested by the CCK-8 assay. Treatment with UK (125 nM–500 nM) for 12 h had no effect on cell viability of BV-2 cells while UK (750 nM–1000 nM) decreased cell viability of BV-2 microglial cells. LPS treatment (125–1000 ng/mL) for 12 h showed no cytotoxicity on BV-2 cells. NS: *p* > 0.05 and ^*∗*^*p* < 0.05 versus the control group.

To examine the cytotoxicity of UK and LPS on BV-2 cells and select the suitable drug concentrations for the subsequent experiments, the effects of UK and LPS on cell viability were detected by the CCK-8 assay. As shown in Figure 1, cell viabilities after treatment with UK (125 nM, 250 nM, and 500 nM) for 12 h had no effect on cell viability of BV-2 cells. However, cell viability of BV-2 cells decreased when the concentration of UK increased to 750 nM and 1000 nM (*p* < 0.05). Meanwhile, LPS treatment at the concentrations at 125–1000 ng/mL for 12 h showed no cytotoxicity on BV-2 cells when compared with the control group (*p* > 0.05). Therefore, the concentrations of 125 nM, 250 nM, and 500 nM of UK and 1000 ng/mL of LPS were selected as the working concentrations for the following experiments.

### 3.2. The Effect of UK on the Expressions of Proinflammatory Cytokines in LPS-Stimulated BV-2 Cells

**Figure 2:**
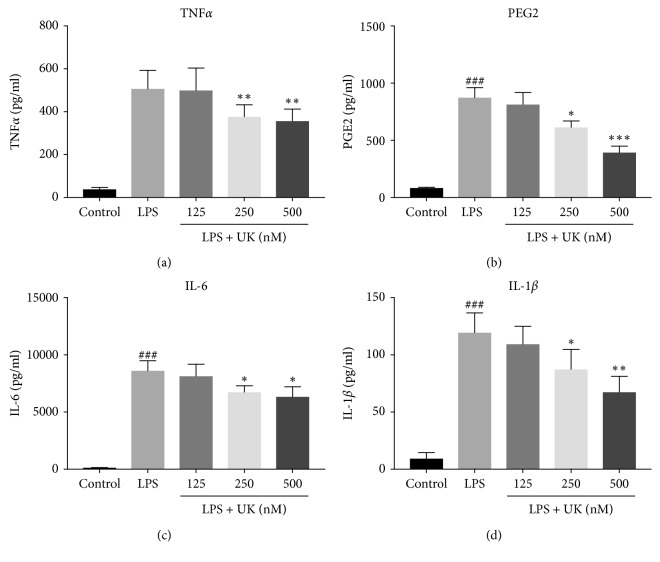
The effect of UK on the expressions of proinflammatory cytokines in LPS- stimulated BV-2 cells. Cells were pretreated with UK (125 nM, 250 nM, and 500 nM) for 12 h and with LPS (1000 ng/mL) treatment for another 12 h. The production of TNF*α*, PGE2, IL-6, and IL-1*β* was measured by ELISA kits. ^###^*p* < 0.001 versus the control group, ^*∗*^*p* < 0.05 versus the LPS-stimulated group, ^*∗∗*^*p* < 0.01 versus the control group, and ^*∗∗∗*^*p* < 0.001 versus the LPS-stimulated group, *n* = 3.

**Figure 3:**
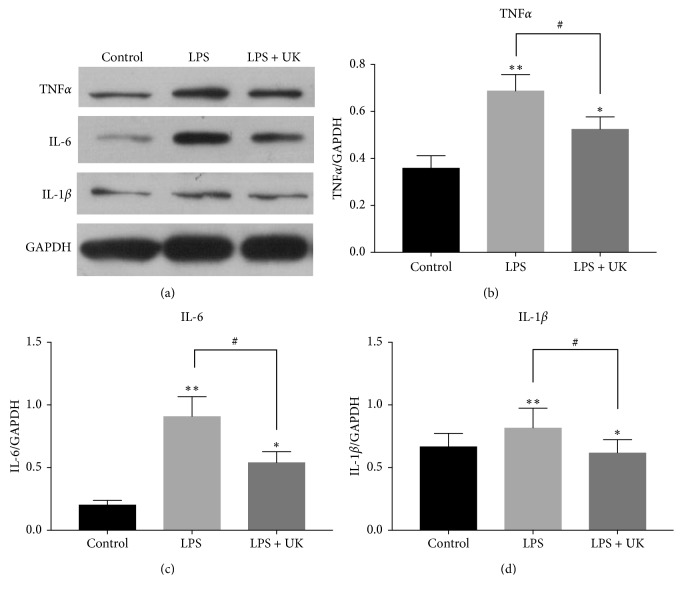
The effect of UK on the protein expressions of proinflammatory cytokines by western blot analysis. Cells were pretreated with UK (500 nM) for 12 h and with LPS (1000 ng/mL) treatment for another 12 h. The production of TNF*α*, IL-6, and IL-1*β* was analyzed by western blot. ^#^*p* < 0.05 versus the LPS-stimulated group, ^*∗*^*p* < 0.05 versus the control group, and ^*∗∗*^*p* < 0.01 versus the control group, *n* = 3.

TNFα, PGE2, IL-6, and IL-1β are proinflammatory cytokines which have been demonstrated in the process of migraine. As a result, the productions of TNF*α*, PGE2, IL-6, and IL-1*β* were measured by ELISA kits to evaluate the potential anti-inflammatory effects of UK on LPS-stimulated BV-2 microglial cells. Cells were pretreated with UK (125 nM, 250 nM, and 500 nM) for 12 h and with LPS (1000 ng/mL) treatment for another 12 h. As shown in Figure 2, LPS administration increased the levels of TNF*α*, PGE2, IL-6, and IL-1*β* when compared with the control group (*p* < 0.05). However, the productions of these cytokines decreased significantly upon introduction with UK at the concentration of 250 nM (*p* < 0.05) and 500 nM (*p* < 0.05), even though there was no significant change at the concentration of 125 nM (*p* > 0.05). In order to confirm the effect of UK on protein expressions of proinflammatory cytokines, the production of TNF*α*, IL-6, and IL-1*β* was analyzed by western blot. Cells were pre-treated with UK (500 nM) for 12 h and with LPS (1000 ng/mL) treatment for another 12 h. Consistently, our results showed that LPS increased the protein levels of TNF*α*, IL-6, and IL-1*β* (*p* < 0.05) and UK treatment decreased the upregulation of protein levels induced by LPS (*p* < 0.05) (Figure 3). Taken together, our results indicated that UK showed anti-inflammatory capacity in a dose-dependent manner in LPS-induced BV-2 cells.

### 3.3. UK Reduced Oxidative Stress in LPS-Induced BV-2 Cells

**Figure 4:**
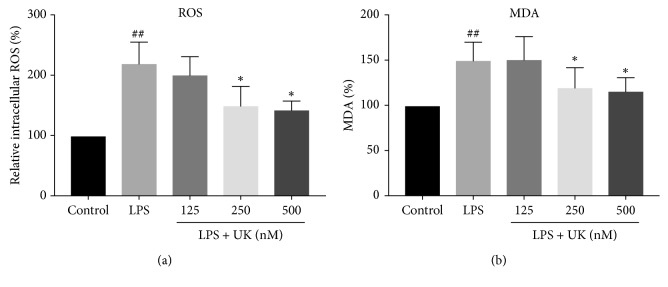
The effect of UK on oxidative stress in LPS-induced BV-2 cells. BV-2 cells were pretreated with UK (125 nM, 250 nM, and 500 nM) for 12 h and with LPS (1000 ng/mL) treatment for another 12 h. ROS level was examined by the DCFH-DA assay, and the production of MDA was determined by MDA kits. ^##^*p*<0.01 versus the control group and ^*∗*^*p* < 0.05 versus the LPS-stimulated group.

Multiple pieces of literature have shown that oxidative stress plays a vital role in migraine. As a result, intracellular ROS level was examined by the DCFH-DA assay and the production of MDA was determined by MDA kits in the present study. Our results showed that intracellular ROS was increased after LPS treatment in BV-2 cells (Figure 4) (*p* < 0.05). Additionally, lipid peroxidation marker MAD level also increased after LPS stimulation (Figure 4) (*p* < 0.05). Notably, a significant reduction in both intracellular ROS and MAD levels was observed after pretreated with UK (250 nM and 500 nM) for 12 h. The concentration of 125 nM of UK had no effect on LPS-induced BV-2 cells in terms of either intracellular ROS or MAD level (*p* > 0.05). Our data indicate that the effect of LPS on the intracellular ROS and MAD levels in BV-2 cells could be alleviated by UK in a dose-dependent manner.

## 4. Discussion

UK, a tissue kallikrein isolated from human urine, is a widely used drug for the treatment of ischemic stroke in China [11]. However, there is still no evidence for the role of UK play on inflammation and oxidative stress in model of migraine, which is a multifactorial neurodegenerative disease without satisfactory treatment.

UK is a commercially available KKS-regulating medicine, and the safety of UK has been well demonstrated [12]. Consistently, our results showed that cell viabilities of BV-2 cells after treatment with UK (125 nM, 250 nM, and 500 nM) for 12 h had no change when compared with the control group.

Neuroinflammation has been thought to play an important role in migraine [7]. PGE2 and proinflammation cytokines (IL-6, IL-1*β*, and TNF*α*) are crucial indicators of the inflammatory process [18]. In the present study, we observed that UK treatment sufficiently reduced LPS-stimulated neuroinflammation (Figures 2 and 3).

Accumulated evidence has been provided for the role of oxidative stress in migraine [6]. Our study highlighted the inhibition of UK on oxidative stress including intracellular reactive oxygen species and MDA level. Consistently, it has been demonstrated that UK was able to rescue glutamate-induced cell death by attenuating reactive oxygen species production and NOS activity in cultured cortical neurons [19]. The activation of bradykinin B2 receptor (B2R), extracellular signal-regulated kinase 1/2 cascade (ERK1/2), BDNF, and Bcl-2 was thought to be involved in this process. Xia et al. reported that tissue kallikrein gene therapy could protect mouse models from oxidative stress and apoptosis via B2R activation [20]. In addition, B2R-dependent regulation of autophagy is involved in inhibiting oxygen and glucose deprivation-induced neurocytotoxicity [21].

However, the exact mechanism of UK on LPS-induced neuroinflammation in BV-2 cells still remains to be explored. Proinflammatory cytokines are regulated by a transcription factor, NF-*κ*B. It has been proved that UK protected neuron through nuclear factor-kappaB (NF-*κ*B) pathway [9]. Inhibition of NF-*κ*B in the microglia could possibly reduce the expressions of inflammatory cytokines.

The experiment conducted by Yang et al. showed that UK functioned on cerebral ischemia in a rat model by decreasing inflammatory responses [9]. By western blot analysis of the brain tissues, they also found that the levels of TLR4 and NF-*κ*B both significantly reduced after the treatment of UK. They made a conclusion that UK protects ischemic stroke rat model through antioxidation and anti-inflammation by inhibiting NF-*κ*B pathway, which is consistent with our results.

The mechanism of UK on LPS-stimulated BV-2 cells might be related to the transforming growth factor-beta 1 (TGF-*β*1), which can inhibit neuroinflammation. Previous studies proved that UK could upregulate TGF-*β*1 and decrease high-sensitivity c-reactive protein, which activates Bcl-2 expression to suppress the apoptosis [22]. Su et al. reported that UK protected neurons against hypoxia-induced cell injury. The process possibly because UK upregulated the phosphorylation of the ERK1/2 cascades by activating Homer1b/c [23].

LPS-stimulated BV-2 cell is a well-established *in vitro* model for inflammation [13]. As a result, we used BV-2 cells in the present research. However, the effect of UK on migraine remains to be examined in animal model.

## 5. Conclusion

We herein report the effect of UK on inflammatory response and oxidative stress in LPS-induced BV-2 cells, which might be a potential therapy for migraine.

## Data Availability

The data used to support the findings of this study are available from the corresponding author upon request.
